# The long-term reproducibility of the white-coat effect on blood pressure as a continuous variable from the Ohasama Study

**DOI:** 10.1038/s41598-023-31861-9

**Published:** 2023-03-27

**Authors:** Michihiro Satoh, Tomoya Yoshida, Hirohito Metoki, Takahisa Murakami, Yukako Tatsumi, Takuo Hirose, Kyosuke Takabatake, Megumi Tsubota-Utsugi, Azusa Hara, Kyoko Nomura, Kei Asayama, Masahiro Kikuya, Atsushi Hozawa, Yutaka Imai, Takayoshi Ohkubo

**Affiliations:** 1 Division of Public Health, Hygiene and Epidemiology, Faculty of Medicine, Tohoku Medical and Pharmaceutical University, 1-15-1 Fukumuro, Miyagino-Ku, Sendai, Miyagi 983-8536 Japan; 2 Department of Preventive Medicine and Epidemiology, Tohoku Medical Megabank Organization, Tohoku University, Sendai, Japan; 3 Tohoku Institute for Management of Blood Pressure, Sendai, Japan; 4 Division of Aging and Geriatric Dentistry, Department of Rehabilitation Dentistry, Tohoku University Graduate School of Dentistry, Sendai, Japan; 5 Department of Hygiene and Public Health, Teikyo University School of Medicine, Tokyo, Japan; 6 Department of Endocrinology and Applied Medical Science, Tohoku University Graduate School of Medicine, Sendai, Japan; 7 Division of Nephrology and Endocrinology, Faculty of Medicine, Tohoku Medical and Pharmaceutical University, Sendai, Japan; 8 Division of Drug Development and Regulatory Science, Faculty of Pharmacy, Keio University, Tokyo, Japan; 9 Department of Environmental Health Science and Public Health, Akita University Graduate School of Medicine, Akita, Japan

**Keywords:** Cardiovascular diseases, Epidemiology, Diagnosis

## Abstract

There is little information about the reproducibility of the white coat effect, which was treated as a continuous variable. To investigate a long-term interval reproducibility of the white-coat effect as a continuous variable. We selected 153 participants without antihypertensive treatment (men, 22.9%; age, 64.4 years) from the general population of Ohasama, Japan, to assess the repeatedly measured white-coat effect (the difference between blood pressures at the office and home) in a 4-year interval. The reproducibility was assessed by testing the intraclass correlation coefficient (two-way random effect model-single measures). The white-coat effect for systolic/diastolic blood pressure slightly decreased by 0.17/1.56 mmHg at the 4-year visit on average. The Bland–Altman plots showed no significant systemic error for the white-coat effects (*P* ≥ 0.24). The intraclass correlation coefficient (95% confidence interval) of the white-coat effect for systolic blood pressure, office systolic blood pressure, and home systolic blood pressure were 0.41 (0.27–0.53), 0.64 (0.52–0.74), and 0.74 (0.47–0.86), respectively. Change in the white-coat effect was mainly affected by a change in office blood pressure. Long-term reproducibility of the white-coat effect is limited in the general population without antihypertensive treatment. The change in the white-coat effect is mainly caused by office blood pressure variation.

## Introduction

White-coat hypertension is defined as a condition in which office blood pressure (BP), but not out-of-office BP, is elevated. Patients with white-coat hypertension have higher cardiovascular or kidney disease risk than those with true normotension, especially in untreated individuals with older age or at high cardiovascular risk^1–9^. White-coat hypertension tends to develop into out-of-office hypertension^5,10^. Therefore, detecting individuals with white-coat hypertension and following them up can prevent cardiovascular diseases in the long term.

One critical problem in evaluating white-coat hypertension is its reproducibility. According to these previous studies, these hypertension phenotypes are moderately reproducible, especially in short-term intervals and in individuals without antihypertensive treatment^11–21^. However, the issues exist. First, the hypertension phenotype is generally fluctuant in individuals with BP readings close to the hypertension threshold. It is more important to assess the reproducibility of the white-coat effect as a continuous variable. We recently reported that office BP levels were associated with a higher risk of out-of-office hypertension based on self-measured BP at home (home BP) after adjustments by baseline home BP^22^. This implies that the white-coat effect, which is calculated as a continuous variable, has clinical significance in the prediction of future out-of-office BP elevation. Second, there is little information about the reproducibility of the white-coat effect over more than a 1-year interval^13^.

A previous study using multiple ambulatory BP monitoring with a 1.2-year interval reported that hypertension phenotype category, i.e. normotension, white coat hypertension, masked hypertension (only in out-of-office conditions), and sustained hypertension (hypertension both office and out-of-office) was moderately reproducible in 39 untreated patients^13^. The white coat effect for systolic BP measured in a 1.2-year interval showed moderately good reproducibility^13^. Therefore, the reproducibility of the white coat effect may be maintained for approximately one year in untreated individuals. Ambulatory BP, which is influenced by daily activities, was used in a previous study^13^. The white coat effect could be reproducible in the long term by using home BP, which is measured under stable conditions^23,24^.

The Ohasama study has measured home BP for a long term in residents of Ohasama town, a rural community in Japan. Using data from the Ohasama study, the present study examined the 4-year interval reproducibility of the white-coat effect, treated as a continuous variable, and examined the reproducibility of the BP indices with the longest interval in untreated individuals. By clarifying this, it is possible to determine whether the white coat effect is fixed individually or fluctuates over time. If the long-term reproducibility of the white coat effect is good, its frequent evaluation in clinical practice is not necessary. Furthermore, we can assume that the high cardiovascular risk of white coat hypertension could be caused by a consistently high office BP relative to out-of-office BP during follow-up.

## Methods

Because the data have a sensitive nature and are maintained based on the study participants’ agreement, the data and study materials will not be made available to other researchers to reproduce results or replicate the procedure. The present study was reported following the Strengthening the Reporting of Observational Studies in Epidemiology (STROBE) guidelines^25^.

### Study design

This report was part of the Ohasama study, a prospective cohort study that started in 1986. Details of the study, including the socioeconomic and demographic characteristics of this region, have been described previously^6,26^. This study complied with the Declaration of Helsinki, and the Institutional Review Boards of Teikyo University (16-075-7), and Tohoku Medical and Pharmaceutical University approved the study protocol (2022-005 [2022-0-006]).

We performed follow-up examinations of home BP measurements every 4 years for each participant. For the current analyses, the data collected between fiscal years (April to March) 2005 and 2019 were used since only two types of devices were used for each home and office BP measurement in this term. The first visit during this period was defined as the baseline, and the second visit 4 years later was defined as a follow-up examination. Data from the entire eligible population were used.

### Blood pressure (BP) measurements

Public health nurses or study investigators instructed participants to measure their home BP using the Omron HEM-747ICN or HEM7080IC cuff-oscillometric upper arm-cuff BP-monitoring device (Omron Healthcare, Kyoto, Japan)^26–28^. Participants were instructed to measure their home BP for 4 weeks, after ≥ 2 min of rest in the morning within 1 h after awakening, maintaining the arm-cuff position at heart level during rest, and, if applicable, before taking their BP-lowering medications^23,24^. They were also instructed to measure their home evening BP every evening just before going to bed. Participants were not asked to measure their home BP twice or more per occasion^23^. The second measurement value was reportedly lower than the first measurement^24^. Since only some participants in the present study measured BP twice per measurement occasion, the first value of each measurement occasion was uniformly used to eliminate the differences in BP due to the measurement number variation. We defined home BP as the mean of all measurements during each examination period.

Office BP was measured twice by medical staff at a local medical center, after resting for at least 2 min at a sitting posture with the arm-cuff position maintained at the level of the heart, using a semiautomatic BP-measuring device based on the oscillometric method (HEM-907 or HEM-9000AI, Omron Healthcare Co. Ltd., Kyoto, Japan)^29^. The mean of the two measurements was used for analysis.

The primary outcome was the 4-year reproducibility of the white coat effect, defined as the difference between office and home BP. We further defined home hypertension as home systolic BP ≥ 135 and/or home diastolic BP ≥ 85 mmHg, and office hypertension as office systolic BP ≥ 140 and/or office diastolic BP ≥ 90 mmHg according to the hypertension management guidelines^24,30^. Based on these hypertension definitions, we further defined normotension, white-coat hypertension (home BP < 135/ < 85 mmHg and office BP ≥ 140/ ≥ 90 mmHg), masked hypertension (home BP ≥ 135/ ≥ 85 mmHg and office BP < 140/ < 90 mmHg), and sustained hypertension (home BP ≥ 135/ ≥ 85 mmHg and office BP ≥ 140/ ≥ 90 mmHg).

### Other information

We gathered information on smoking status, alcohol consumption, medications, and histories of diseases through a questionnaire survey, medical interview, or reviewing medical records. Diabetes was defined as a random glucose level ≥ 11.1 mmol/L (≥ 200 mg/dL), a fasting glucose level ≥ 7.0 mmol/L (≥ 126 mg/dL), hemoglobin A1c based on the National Glycohemoglobin Standardization Program threshold ≥ 6.5%, or the use of oral antidiabetic drugs or insulin. Dyslipidemia was indicated by low-density lipoprotein cholesterol ≥ 3.62 mmol/L (≥ 140 mg/dL), high-density lipoprotein cholesterol < 1.03 mmol/L (< 40 mg/dL), triglycerides ≥ 1.69 mmol/L (≥ 150 mg/dL), or use of anti dyslipidemia medications.

### Statistical analyses

We selected the participants without antihypertensive treatment both at baseline and the 4-year visit. The characteristics between follow-up and non-follow-up participants were tested to confirm selection bias. The difference in hypertension phenotype between baseline and the 4-year visit was examined by Bowker’s test of symmetry and kappa statistics. The level of agreement was defined based on the κ value as follows: 0–0.20, minimal agreement; 0.21–0.40, fair agreement; 0.41–0.60, moderate agreement; 0.61–0.80, substantial agreement; and 0.81–1.00, almost perfect or perfect agreement^31^. The reproducibility of the white-coat effect as a continuous variable was assessed by testing for association, bias, agreement, and consistency. Pearson’s correlation coefficients and regression equations were calculated to examine the association between the white-coat effect at baseline and that at the 4-year visit. To examine the bias between the measurements, we used the Bland–Altman analysis, which plots the average of the two measurements on the x-axis and the difference between the two measurements on the y-axis^32^. A significant regression slope in the Bland–Altman plot suggests the presence of systematic error in the measurements^32^. Intraclass correlation coefficients (ICCs) were calculated between the baseline and 4-year visit values to assess agreement and consistency between them. The agreement was assessed using ICC from the two-way random effect model single measures, which is generally indicated as ICC (2, 1)^33,34^. The ICC (2, 1) values were lower due to age-related changes in BP indices. The consistency was assessed using ICC from the two-way mixed effect model single measures, or the ICC (3, 1)^33,34^. The ICC (3, 1) model indicates consistency but not an agreement between measurements since it deals with the mean difference between measurements as a systematic error, resulting in ICC values not considering age-related BP changes^33,34^. The value of the ICC ranges from 0 to 1, where one represents the perfect reliability of the measurement. An ICC of ≥ 0.70 is required as a minimum standard for test–retest reliability^35,36^, although there is no established threshold of ICC. We also calculated the ICC for the white-coat effect in the participants who measured their home and office BP using the same devices (HEM-747ICN and HEM-907, respectively) in the baseline and 4-year visits to confirm the effect of differences in measurement devices. To exclude the basic characteristics differences, stratification analysis by sex or age (< 65/ ≥ 65 years) was performed.

Participants were stratified by the difference in white-coat effect for systolic BP between the 4-year visit and baseline < − 10 mmHg, − 10–10 mmHg, and > 10 mmHg. To examine factors associated with a change in the white-coat effect, participant characteristics were compared among these three groups.

Statistical differences in means and proportions were assessed using Welch’s t-test or analysis of variance and Fisher’s exact test, respectively. The office statistical significance was set at an α-level < 0.05 on two-tailed tests. Data were expressed as mean ± standard deviation unless otherwise stated. R software (version 4.2.1, R Foundation for Statistical Computing) was used for the calculation of ICCs, and SAS version 9.4 (SAS Institute, Cary, NC, USA) was used for other statistical analyses.

## Results

### Participant selection

**Figure 1:**
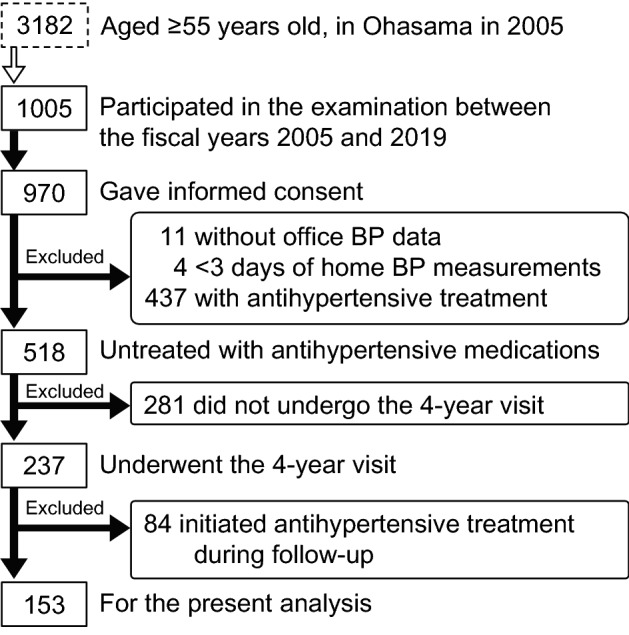
Flowchart for participant selection.

In 2005, 3182 individuals aged ≥ 55 years lived in Ohasama. Figure 1 shows the flowchart of participant selection. Of those, 1005 participated in the examination between the fiscal years 2005 and 2019, and 970 provided informed consent to participate in this study. We excluded 11 participants without office BP data and 4 who did not measure their home BP for ≥ 3 days. We excluded 437 patients under antihypertensive treatment at baseline to exclude the effect of the antihypertensive regimen change. For this analysis, 281 participants were excluded because they did not undergo the 4-year follow-up visit. Among them, those excluded because of loss to follow-up had a higher proportion of alcohol drinkers relative to the 237 participants who underwent the follow-up; there were no significant differences in BP levels between these two groups (Supplementary Table S1). Of the remaining 237 participants, 14.4% (n = 19/132) with normotension, 41.0% (n = 16/39) with white-coat hypertension, 65.2% (n = 15/23) with masked hypertension, and 79.1% (n = 34/43) with sustained hypertension experienced antihypertensive treatment initiation; these 84 participants who initiated antihypertensive treatment between baseline and the 4-year visit were also excluded (baseline characteristics in 84 individuals are shown in Supplementary Table S2). Finally, 153 participants, who were not treated with antihypertensive drugs both at baseline and the 4-year visit, were included in the present analysis. Home and office systolic/diastolic BP levels at baseline were higher in 84 participants excluded due to antihypertensive treatment initiation than in 153 untreated participants (all *P* < 0.0001).

### Participant characteristics

The characteristics of 153 participants at baseline and the 4-year visit are shown in Table 1. From baseline, body mass index and the proportion of alcohol drinkers decreased at the 4-year visit. Office systolic and home systolic/diastolic BP increased. The white-coat effect for diastolic BP significantly decreased, whereas the amplitude of change was small.

**Table 1:** Participants’ characteristics at baseline and 4-year visit.

Variable	At baseline	At 4-year visit	Paired test *P*
Men, %	22.9	22.9	–
Age, years	64.4 ± 5.8	68.4 ± 5.8	< 0.0001
BMI, kg/m^2^	23.1 ± 3.2	22.9 ± 3.2	0.045
Current smoking, %	8.5	5.9	0.16
Alcohol consumption, %	37.9	25.5	0.0009
Diabetes, %	3.3	4.6	0.32
Dyslipidemia, %	54.2	54.9	> 0.99
History of CVD, %	7.2	7.2	> 0.99
Systolic BP, mmHg			
Office	127.5 ± 15.8	132.0 ± 18.2	< 0.0001
Home	120.3 ± 11.3	125.0 ± 11.7	< 0.0001
White-coat effect (office-home)	7.2 ± 12.8	7.0 ± 13.9	0.88
Diastolic BP, mmHg			
Office	73.0 ± 9.5	72.7 ± 10.1	0.61
Home	71.8 ± 7.0	73.1 ± 7.4	0.0008
White-coat effect (office-home)	1.2 ± 7.7	– 0.4 ± 7.8	0.024
Pulse rate, bpm			
Office	68.4 ± 9.5	68.4 ± 9.5	0.97
Home	64.3 ± 7.0	64.8 ± 7.3	0.14

*BMI* body mass index, *CVD* cardiovascular disease, *BP* blood pressure.

### Reproducibility of hypertension phenotypes

The prevalence of normotension, white-coat hypertension, masked hypertension, and sustained hypertension at baseline was 113 (73.9%), 23 (15.0%), 8 (5.2%), and 9 (5.9%), respectively. Of those, 103 (67.3%) participants had the same hypertension phenotype at baseline and the 4-year visit. Of the 23 participants with white-coat hypertension, 4 and 8 participants, switched to the normotension or sustained hypertension group at the 4-year visit, respectively (characteristics based on the hypertension phenotype at the 4-year visit are shown in Supplementary Table S3). The agreement between hypertension phenotypes at baseline and the 4-year visit was minimal (Table 2) (κ = 0.36; 95% confidence interval, 0.25–0.48; χ^2^ for symmetry test = 17.4; df = 6; *P* = 0.0079).

**Table 2:** Agreement among hypertension phenotypes.

	*Normotension at* the 4-year visit	*White-coat HT at* the 4-year visit	*Masked HT at* the 4-year visit	*Sustained HT at* the 4-year visit
Hypertension phenotypes at baseline				
Normotension (n = 113), n (%)	86 (76.1)	14 (12.4)	8 (7.1)	5 (4.4)
White-coat HT (n = 23), n (%)	4 (17.4)	11 (47.8)	0 (0.0)	8 (34.8)
Masked HT (n = 8), n (%)	2 (25.0)	2 (25.0)	2 (25.0)	2 (25.0)
Sustained HT (n = 9), n (%)	1 (11.1)	1 (22.2)	2 (22.2)	4 (44.4)

*HT* hypertension.

### Reproducibility of difference between office and home BP as a continuous variable

**Figure 2:**
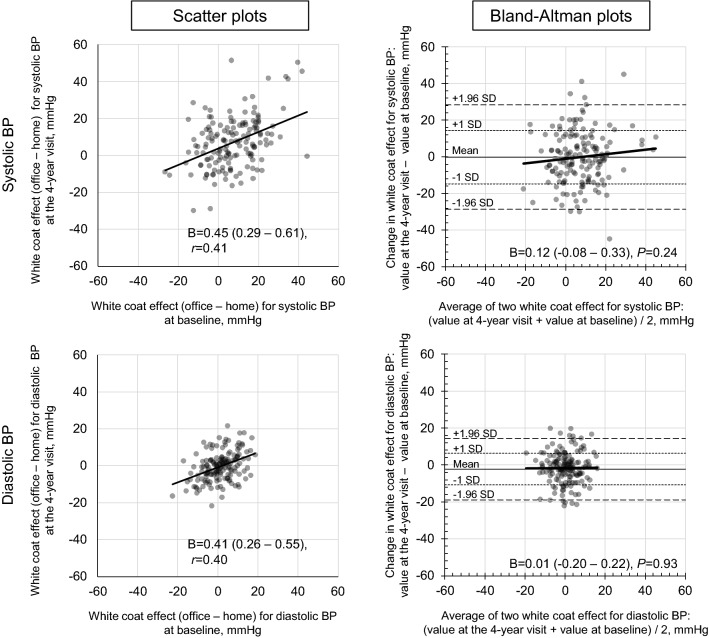
Scatterplots and Bland–Altman plots between the white-coat effect at baseline and the 4-year visit. “Β” and “r” refer to the regression coefficient (95% confidence interval) and Pearson’s correlation coefficient, respectively. *BP* blood pressure**.**

The white-coat effects at baseline were moderately correlated with those at the 4-year visit (*r* = 0.41 for systolic and *r* = 0.40 for diastolic) (Fig. 2). The regression coefficients of the white-coat effects at baseline against the values at the 4-year visit were 0.45 for systolic and 0.41 for diastolic (Fig. 2). No significant regression slopes were observed in the Bland–Altman plots (*P* = 0.24 for systolic and *P* = 0.93 for diastolic) (Fig. 2).

The white-coat effect for systolic/diastolic BP decreased by 0.17/1.56 mmHg at the 4-year visit. The ICCs of the white-coat effect for systolic and diastolic BPs were 0.41 and 0.40, respectively; those were similar in the analysis based on 94 participants who measured their BP using the same office and home measurement devices (Table 3). The reproducibility was good (ICC > 0.7) only for home BP (Table 3). When home evening BP was used to define the white-coat effect instead of home morning BP, the results were similar to those based on home morning BP: the ICCs (95% confidence intervals) of the white-coat effect for systolic and diastolic BPs were 0.47 (0.34–0.59) and 0.44 (0.30–0.56), respectively (Supplementary Table S4). When home morning BP measured only for 7 days was used, the ICCs (3, 1) of the white-coat effect for systolic and diastolic BPs decreased to 0.34 and 0.35, respectively (Supplementary Table S5).

**Table 3:** Reproducibility of the white coat effect and BP levels.

	All participants (n = 153)			With the same BP devices* (n = 94)		
	Amplitude of change, mmHg (4-year—baseline)	Agreement: ICC (95% CI)	Consistency: ICC (95% CI)	Amplitude of change, mmHg (4-year—baseline)	Agreement: ICC (95% CI)	Consistency: ICC (95% CI)
White-coat effect for systolic BP	− 0.17 ± 14.53	0.41 (0.27–0.53)	0.41 (0.27–0.53)	− 1.31 ± 15.09	0.45 (0.27–0.60)	0.45 (0.27–0.60)
White-coat effect for diastolic BP	− 1.56 ± 8.47	0.40 (0.26–0.52)	0.40 (0.26–0.53)	− 1.46 ± 8.68	0.34 (0.15–0.51)	0.35 (0.16–0.51)
Home systolic BP	4.68 ± 7.23	0.74 (0.47–0.86)	0.80 (0.74–0.85)	4.94 ± 7.50	0.71 (0.41–0.84)	0.78 (0.68–0.85)
Home diastolic BP	1.23 ± 4.45	0.80 (0.72–0.85)	0.81 (0.75–0.86)	1.04 ± 4.52	0.78 (0.68–0.85)	0.78 (0.69–0.85)
Office systolic BP	4.51 ± 13.94	0.64 (0.52–0.74)	0.67 (0.57–0.75)	3.63 ± 14.78	0.62 (0.48–0.73)	0.63 (0.49–0.74)
Office diastolic BP	− 0.33 ± 7.95	0.67 (0.58–0.75)	0.67 (0.58–0.75)	− 0.43 ± 8.31	0.58 (0.42–0.70)	0.58 (0.42–0.70)

Agreement and consistency were assessed by ICC from the two-way random effect model single measures (ICC [2, 1]) and two-way mixed effect model single measures (ICC [3, 1]), respectively.

*BP* blood pressure, *ICC* intraclass correlation coefficient, *CI* confidence interval.

*The participants who used HEM-747ICN for home BP measurements and HEM-907 for office BP measurements were included in this analysis.

The stratification analyses according to sex (Supplementary Table S6) or age (Supplementary Table S7) were performed. The point estimates of ICC for the white coat effect ranged from 0.32 to 0.52. The ICC levels for home BP were consistently higher than those for office BP. The ICC (2, 1) and ICC (3, 1) for the white coat effect for systolic BP were 0.17 (− 0.03–0.36) and 0.19 (− 0.03–0.39), respectively, in the 84 participants excluded due to antihypertensive treatment initiation.

### Factors associated with the change in the magnitude of the white-coat effect

The white-coat effect for systolic BP at baseline was inversely associated with the 4-year change in the white-coat effect (Table 4). The difference in office systolic BP strongly contributed to the large change in the white-coat effect; for instance, in the participants with ≤ − 10 mmHg change (decreased at the 4-year visit) in the white-coat effect for systolic BP, the office systolic BP decreased by 11.3 mmHg (134.2–122.9 mmHg), whereas the home systolic BP increased only by 7.1 mmHg (119.1–126.2 mmHg) (Table 4). The participants’ characteristics other than BP levels were not associated with the change in the white-coat effect (Table 4).

**Table 4:** Characteristics according to the white-coat effect.

	Change in the white-coat effect for systolic BP (the 4-year visit − baseline), mmHg			*P*
	≤ − 10 (n = 38)	> − 10, < 10 (n = 75)	≥ 10 (n = 40)	
Men, %	26.3	22.7	20.0	0.82
Age, years	64.8 ± 5.9	63.7 ± 5.6	65.3 ± 6.3	0.36
BMI, kg/m^2^	23.6 ± 3.6	22.6 ± 3.0	23.4 ± 3.1	0.27
Change in BMI, kg/m^2^	– 0.5 ± 1.0	0.0 ± 1.1	– 0.1 ± 1.0	0.12
Current smoking, %	13.2	6.7	7.5	0.48
Stop smoking at the 4-year visit, %	2.6	4.0	5.0	> 0.99
Alcohol consumption, %	34.2	40.0	37.5	0.84
Stop drinking at the 4-year visit, %	15.8	18.7	15.0	0.89
Diabetes, %	5.3	2.7	2.5	0.72
Dyslipidemia, %	55.3	54.7	52.5	0.95
History of CVD, %	5.3	6.7	10.0	0.78
Systolic BP, mmHg				
Office at baseline	134.2 ± 16.7	126.6 ± 14.1	122.8 ± 16.2	0.0044
Home at baseline	119.1 ± 12.9	118.4 ± 10.0	124.8 ± 11.0	0.012
White-coat effect at baseline	15.0 ± 10.6	8.1 ± 10.9	– 2.0 ± 12.4	< 0.0001
Office at the 4-year visit	122.9 ± 17.1	132.0 ± 16.4	140.6 ± 18.6	< 0.0001
Home at the 4-year visit	126.2 ± 11.6	124.5 ± 11.9	124.7 ± 11.5	0.75
White-coat effect at the 4-year visit	– 3.3 ± 10.5	7.5 ± 12.2	15.9 ± 13.4	< 0.0001
Diastolic BP at baseline, mmHg				
Office at baseline	75.8 ± 8.8	72.9 ± 8.9	70.4 ± 10.5	0.045
Home at baseline	70.5 ± 6.9	71.2 ± 6.8	74.3 ± 7.0	0.028
White-coat effect at baseline	5.3 ± 6.9	1.8 ± 6.7	– 3.9 ± 7.6	< 0.0001
Office at the 4-year visit	69.0 ± 9.7	73.6 ± 9.3	74.3 ± 11.4	0.037
Home at the 4-year visit	72.4 ± 7.0	73.2 ± 7.8	73.4 ± 7.2	0.83
White-coat effect at the 4-year visit	– 3.4 ± 8.5	0.4 ± 6.9	0.9 ± 8.1	0.023
Pulse rate, bpm				
Office at baseline	69.5 ± 9.0	67.8 ± 9.5	68.4 ± 9.9	0.65
Home at baseline	64.0 ± 5.6	64.1 ± 7.8	65.0 ± 6.7	0.77
Office at the 4-year visit	69.3 ± 8.9	68.2 ± 9.8	67.8 ± 9.4	0.76
Home at the 4-year visit	64.6 ± 5.7	64.8 ± 8.0	65.1 ± 7.3	0.95

*BMI* body mass index, *CVD* cardiovascular disease, *BP* blood pressure.

## Discussion

The reproducibility of hypertension phenotypes with a 4-year interval was considered to be minimal to fair from the kappa values. The long-term reproducibility of the white-coat effect, which was used as a continuous variable, was also limited since the ICC value did not reach 0.7, although no significant systemic errors were confirmed. The variability of the white-coat effect change was mainly caused by the large office BP difference.

The reproducibility of hypertension phenotypes was limited when it was reassessed after 4 years (κ-value, 0.36). This is possibly attributed to the high proportion of normotension (73.9% were normotensives in the untreated participants). A previous meta-analysis reported a similar κ-value (0.39) regarding hypertension phenotype reproducibility based on home BP in untreated individuals^21^. This meta-analysis included the studies conducted within 1 week or half a year^21^. Therefore, the hypertension phenotype reproducibility appeared not to be good regardless of the measurement interval.

One important issue in the evaluation of hypertension phenotype is that hypertension classification easily changes in individuals with BP readings close to the hypertension threshold. The present findings suggest that the white-coat effect is not reproducible even when it is assessed as a continuous variable. The ICC between white-coat effects at baseline and the 4-year visit was approximately 0.4 and showed no good reproducibility of white-coat effects in the present study. Only half of the participants had the 4-year change of white coat effect for systolic BP within |10| mmHg. The limited reproducibility of the white-coat effect or hypertension phenotype can cause inconsistent results regarding its prognosis^1–9^. A previous study revealed that the test–retest correlation coefficient between the white coat effects for systolic BP measured in a 1.2-year interval was 0.69 in untreated individuals^13^. The white coat effect may be reproducible within 1 year.

The decrease in the white-coat effect was observed in the participants with a high white-coat effect at baseline, and vice versa. This result can suggest the presence of “regression to the mean” phenomena. The variability of the white-coat effect change appeared to be mainly caused by office BP variation. The previous study assessed the reproducibility of office BP strictly measured by an automated measurement device^37^. Consequently, the variability in office BP between visits was large even in the absence of an observer (unattended office BP) in 287 outpatients^37^. Considering this fact^37^, it could be difficult to capture stable white-coat phenomena. Meanwhile, the reproducibility or repeatability of home BP is reported to be good at least in the 1-year interval^37–39^. The present study expanded this evidence into the longer term.

Apart from the reproducibility issue, the present results also imply that individuals with hypertension cannot easily move to the normotension group. Only 11.1–25.0% of individuals moved to the normotension group among the participants having hypertension. The proportion of antihypertensive treatment initiation was high in the hypertensive participants. If individuals with untreated hypertension are found in a health checkup, we should follow-up with these individuals carefully or should consider initiating treatment.

The strength of the study was the assessment of the long-term reproducibility of the white coat effect as a continuous variable in a general population without antihypertensive treatment. It is difficult to assess an individual’s white coat effect or home BP values in a 4-year interval. White coat hypertension is associated with a long-term, but not short-term, cardiovascular risk^40^. However, from the limited long-term reproducibility of the white coat effect as per the present findings, the white coat phenomenon can change during the long-term follow-up period and can merely be a temporal condition at baseline. This could not be proven in a short-term study. Notably, in clinical practice, the white-coat effect has variability and should be confirmed at least in a 1-year interval^13^. We previously suggested that an “introverted” personality is associated with white-coat hypertension^41^. Future research considering personality is needed to clarify which individuals consistently reveal white coat hypertension in a long term based on a larger sample size.

The present study has some limitations. First, since the present study was conducted on the middle-aged or elderly population from the Japanese rural area, the generalizability and transportability of the findings may be limited. However, the higher reproducibility of home BP than that of office BP was observed similar to the results from the previous studies^37–39^. Third, participants who newly received antihypertensive medications based on each hypertension subtype were excluded. The present results can only be generalized to individuals who do not require antihypertensive treatment. The exclusion of the participants with antihypertensive treatment initiation could affect the reproducibility of the hypertension phenotype and may have underestimated the persistence of hypertension subtypes. Furthermore, we did not assess adherence to antihypertensive treatment in treated participants. Detailed information on the antihypertensive treatment regimen is missing for a proportion of treated participants. For these reasons, we could not assess the accurate reproducibility among the treated participants. The white coat effect in treated patients varies depending on the number of antihypertensive medications or the timing of pill administration^42^. A previous study reported that the Pearson correlation coefficients for the white-coat effect taken 3 months apart were 0.22 for systolic and 0.25 for diastolic pressure in treated patients with resistant hypertension, suggesting poor reproducibility of white-coat hypertension in treated individuals^20^. Fourth, the exclusion of those who were lost to follow-up (n = 281) may have contributed to the poor reproducibility of the white coat effect. However, the selection bias due to loss to follow-up may be limited since large differences in baseline BP levels between the follow-up and non-follow-up participants were not there. Fifth, we used the first value of each measurement occasion for home BP whereas the recent guidelines recommend two measurements on each occasion^24,30,43,44^. The reproducibility of home BP could have been underestimated in the present study. Finally, we do not have data on physical activity, which is known to affect BP levels^24,30,44^.

In conclusion, the long-term reproducibility of the white-coat effect is limited in the general population without antihypertensive treatment. It is difficult to capture a stable white-coat effect in the long term. The change in the white-coat effect includes the regression to the mean phenomenon, which is mainly caused by office BP variation. Since the white-coat effect is fluctuant, home and office BPs should be measured regularly.

## Supplementary Information

Supplementary Tables.

## Data Availability

Because the data have a sensitive nature and are maintained based on the research participants’ agreement, the data and study materials will not be made available to other researchers to reproduce results or replicate the procedure.
