# Supplementary material for: The long-term reproducibility of the white-coat effect on blood pressure as a continuous variable from the Ohasama Study
Source: Sci Rep. 2023 Mar 27;13:4985. doi: 10.1038/s41598-023-31861-9 (PMC10043024; doi:10.1038/s41598-023-31861-9)
Supplement: Supplementary file 1 — Supplementary Tables. [file 41598_2023_31861_MOESM1_ESM.pdf]

Supplementary File

This Online-Only Data Supplement has been provided by the authors to provide readers additional information about the work.

Supplement to:

The long-term reproducibility of the white-coat effect on blood pressure as a continuous variable from the Ohasama Study

Table S1. Characteristics at baseline according to the follow-up status in untreated participants

	Followed (n=237)	Unfollowed (n=281)	*P*
Men, %	28.7	32.0	0.44
Age, years	65.2±6.3	65.5±7.4	0.55
BMI, kg/m^2	23.5±3.2	23.7±3.1	0.57
Current smoking, %	10.1	9.6	0.88
Alcohol consumption, %	43.5	30.6	0.0026
Diabetes, %	6.8	10.3	0.16
Dyslipidemia, %	56.1	57.3	0.79
History of CVD, %	7.6	6.8	0.73
Systolic BP at baseline, mmHg			
Office	133.0±17.3	134.8±18.5	0.27
Home	126.4±13.8	127.9±13.7	0.19
White-coat effect (office − home)	6.7±14.1	6.8±15.6	0.91
Diastolic BP at baseline, mmHg			
Office	75.7±10.6	75.8±11.1	0.92
Home	75.0±8.4	76.0±8.6	0.17
White-coat effect (office − home)	0.7±8.5	-0.2±8.8	0.22
Pulse rate, bpm			
Office	67.9±9.4	68.9±9.8	0.24
Home	63.9±7.6	64.7±7.1	0.18

BMI, body mass index; CVD, cardiovascular disease; BP, blood pressure

Table S2. Characteristics at baseline in participants in whom antihypertensive treatment was initiated before the 4-year period

	Antihypertensive treatment initiated (n=84)
Men, %	39.3
Age, years	66.6±6.7
BMI, kg/m^2	24.2±3.0
Current smoking, %	13.1
Alcohol consumption, %	53.6
Diabetes, %	13.1
Dyslipidemia, %	59.5
History of CVD, %	8.3
Systolic BP at baseline, mmHg	
Office	143.2±15.4
Home	137.5±10.6
White-coat effect (office − home)	5.7±16.2
Diastolic BP at baseline, mmHg	
Office	80.6±10.7
Home	80.7±7.6
White-coat effect (office − home)	-0.1±9.7
Pulse rate, bpm	
Office	67.0±9.4
Home	63.0±8.6

BMI, body mass index; CVD, cardiovascular disease; BP, blood pressure

Table S3. Characteristics of participants with white coat hypertension

	Hypertension phenotype at the 4-year visit			*P*
	NT (n=4)	WCHT (n=11)	SHT (n=8)	
Men, %	0.0	27.3	25.0	0.66
Age, years	69.4±4.7	65.1±4.8	66.4±6.2	0.39
BMI, kg/m^2	25.2±2.4	24.1±3.3	23.4±3.9	0.68
Change in BMI, kg/m^2	-0.1±1.5	-0.1±1.3	-0.2±0.6	0.98
Current smoking, %	0.0	9.1	12.5	>0.99
Stop smoking at the 4-year visit, %	0.0	9.1	0.0	>0.99
Alcohol consumption, %	0.0	63.6	12.5	0.027
Stop drinking at the 4-year visit, %	0.0	27.3	0.0	0.25
Diabetes, %	25.0	9.1	0.0	0.43
Dyslipidemia, %	25.0	72.7	50.0	0.25
History of CVD, %	0.0	0.0	12.5	0.52
Systolic BP, mmHg				
Office at baseline	145.9±6.6	146.0±4.5	154.3±11.5	0.081
Home at baseline	125.9±5.8	123.5±8.0	128.3±4.4	0.32
White-coat effect at baseline	20.0±3.4	22.5±8.8	26.0±11.1	0.53
Office at the 4-year visit	123.4±11.4	154.4±11.6	152.4±14.6	0.0013
Home at the 4-year visit	128.8±3.6	128.2±4.4	138.8±2.2	<0.0001
White-coat effect at the 4-year visit	-5.4±7.9	26.2±10.7	13.6±16.2	0.0012
Diastolic BP at baseline, mmHg				
Office at baseline	80.3±7.7	83.7±7.3	80.1±10.1	0.61
Home at baseline	74.8±5.2	74.7±5.1	74.7±5.6	>0.99
White-coat effect at baseline	5.4±7.6	9.0±6.0	5.4±8.1	0.50
Office at the 4-year visit	68.6±7.0	83.1±7.6	79.1±11.9	0.046
Home at the 4-year visit	73.7±9.5	75.8±4.8	77.7±4.6	0.53
White-coat effect at the 4-year visit	-5.1±7.7	7.3±6.8	1.4±9.4	0.038
Pulse rate, bpm				
Office at baseline	77.9±15.7	69.5±11.9	70.9±9.2	0.48
Home at baseline	65.0±4.9	63.5±7.8	63.3±8.5	0.93
Office at the 4-year visit	74.5±2.6	64.6±6.6	70.3±12.3	0.14
Home at the 4-year visit	66.8±7.5	61.6±6.3	63.4±4.6	0.35

NT, normotension; WCHT, white coat hypertension; SHT, sustained hypertension; BMI, body mass index; CVD, cardiovascular disease; BP, blood pressure

Table S4. Reproducibility of the white-coat effect based on home evening BP instead of home morning BP

	All participants (n=153)			With the same BP devices* (n=94)		
	Amplitude of change, mmHg (4-year – baseline)	Agreement: ICC (95%CI)	Consistency: ICC (95%CI)	Amplitude of change, mmHg (4-year – baseline)	Agreement: ICC (95%CI)	Consistency: ICC (95%CI)
White-coat effect for systolic BP	1.09±15.17	0.47 (0.34–0.59)	0.47 (0.34–0.59)	0.61±15.78	0.48 (0.31–0.62)	0.48 (0.31–0.62)
White-coat effect for diastolic BP	−0.69±8.82	0.44 (0.30–0.56)	0.44 (0.30–0.56)	−0.35±8.74	0.44 (0.26–0.59)	0.44 (0.26–0.59)
Home systolic BP	3.42±7.79	0.74 (0.60–0.82)	0.77 (0.70–0.83)	3.03±8.23	0.69 (0.54–0.79)	0.71 (0.60–0.80)
Home diastolic BP	0.36±4.95	0.77 (0.70–0.83)	0.77 (0.70–0.83)	−0.08±5.12	0.72 (0.61–0.81)	0.72 (0.61–0.81)
Office systolic BP	4.51±13.94	0.64 (0.52–0.74)	0.67 (0.57–0.75)	3.63±14.78	0.62 (0.48–0.73)	0.63 (0.49–0.74)
Office diastolic BP	−0.33±7.95	0.67 (0.58–0.75)	0.67 (0.58–0.75)	−0.43±8.31	0.58 (0.42–0.70)	0.58 (0.42–0.70)

Home evening BP was used to calculate the white-coat effect instead of home morning BP. The values for office BP levels are the same as those in Table 3 since the same participants were included.
*The participants who used HEM-747ICN for home BP measurements and HEM-907 for office BP measurements were included in this analysis.

BP, blood pressure; ICC, intraclass correlation coefficient; CI, confidence interval

Table S5. Reproducibility of the white-coat effect based on home morning BP measured for 7 days

	Amplitude of change, mmHg (4-year – baseline)	Agreement: ICC (95%CI)	Consistency: ICC (95%CI)
White-coat effect for systolic BP	-0.12±15.82	0.34 (0.19–0.47)	0.34 (0.19–0.47)
White-coat effect for diastolic BP	-1.54±9.25	0.34 (0.20–0.48)	0.35 (0.20–0.48)
Home systolic BP	4.63±9.41	0.69 (0.51–0.80)	0.74 (0.65–0.80)
Home diastolic BP	1.21±5.78	0.72 (0.63–0.79)	0.72 (0.64–0.79)
Office systolic BP	4.51±13.94	0.64 (0.52–0.74)	0.67 (0.57–0.75)
Office diastolic BP	−0.33±7.95	0.67 (0.58–0.75)	0.67 (0.58–0.75)

Home morning BP measured for 7 days was used to calculate the white-coat effect. The values for office BP levels are the same as those in Table 3 since the same participants were included.

BP, blood pressure; ICC, intraclass correlation coefficient; CI, confidence interval

Table S6. Reproducibility of white-coat effect based on home morning BP by sex

	Men (n=35)			Women (n=118)		
	Amplitude of change, mmHg (4-year – baseline)	Agreement: ICC (95%CI)	Consistency: ICC (95%CI)	Amplitude of change, mmHg (4-year – baseline)	Agreement: ICC (95%CI)	Consistency: ICC (95%CI)
White-coat effect for systolic BP	-1.68±11.93	0.52 (0.24–0.73)	0.52 (0.24–0.73)	0.27±15.23	0.39 (0.22–0.53)	0.39 (0.22–0.53)
White-coat effect for diastolic BP	1.05±7.99	0.45 (0.14–0.67)	0.45 (0.14–0.68)	-2.33±8.49	0.39 (0.23–0.53)	0.41 (0.25–0.55)
Home systolic BP	6.06±6.90	0.62 (0.11–0.86)	0.74 (0.54–0.86)	4.27±7.30	0.77 (0.55–0.87)	0.81 (0.74–0.87)
Home diastolic BP	1.32±4.95	0.72 (0.52–0.85)	0.73 (0.53–0.86)	1.20±4.31	0.82 (0.74–0.87)	0.83 (0.76–0.88)
Office systolic BP	4.39±11.78	0.65 (0.40–0.81)	0.67 (0.44–0.82)	4.55±14.56	0.65 (0.51–0.75)	0.66 (0.55–0.75)
Office diastolic BP	2.37±7.07	0.68 (0.45–0.83)	0.70 (0.48–0.84)	-1.13±8.05	0.67 (0.56–0.76)	0.68 (0.56–0.76)

BP, blood pressure; ICC, intraclass correlation coefficient; CI, confidence interval

Table S7. Reproducibility of the white-coat effect based on home morning BP by age

	Age<65 (n=85)			Age≥65 (n=68)		
	Amplitude of change, mmHg (4-year – baseline)	Agreement: ICC (95%CI)	Consistency: ICC (95%CI)	Amplitude of change, mmHg (4-year – baseline)	Agreement: ICC (95%CI)	Consistency: ICC (95%CI)
White-coat effect for systolic BP	-0.16±12.88	0.44 (0.25–0.60)	0.44 (0.25–0.60)	-0.19±16.45	0.38 (0.16–0.57)	0.38 (0.16–0.57)
White-coat effect for diastolic BP	-1.40±7.74	0.47 (0.29–0.62)	0.48 (0.29–0.62)	-1.76±9.36	0.32 (0.09–0.52)	0.32 (0.09–0.52)
Home systolic BP	4.90±6.83	0.76 (0.43–0.88)	0.83 (0.75–0.88)	4.41±7.74	0.70 (0.45–0.83)	0.75 (0.63–0.84)
Home diastolic BP	1.56±4.13	0.85 (0.75–0.90)	0.86 (0.79–0.91)	0.81±4.82	0.70 (0.56–0.80)	0.70 (0.56–0.81)
Office systolic BP	4.74±11.91	0.73 (0.58–0.82)	0.75 (0.64–0.83)	4.22±16.21	0.52 (0.32–0.68)	0.53 (0.34–0.68)
Office diastolic BP	0.16±7.38	0.76 (0.78–0.91)	0.76 (0.78–0.91)	-0.95±8.63	0.51 (0.31–0.67)	0.51 (0.31–0.67)

BP, blood pressure; ICC, intraclass correlation coefficient; CI, confidence interval
